# Cumulative evidence for relationships between multiple variants of HNF1B and the risk of prostate and endometrial cancers

**DOI:** 10.1186/s12881-018-0640-7

**Published:** 2018-07-27

**Authors:** Yu Tong, Yi Qu, Shiping Li, Fengyan Zhao, Yibin Wang, Dezhi Mu

**Affiliations:** 1 Department of Pediatrics, West China Second University Hospital, Sichuan University, No.17, Section 3, Renmin Nan Road, Chengdu, 610041 Sichuan Province China; 2 Key Laboratory of Obstetric & Gynecologic and Pediatric Diseases and Birth Defects of Ministry of Education, West China Second University Hospital, Sichuan University, No.17, Section 3, Renmin Nan Road, Chengdu, 610041 Sichuan Province China

**Keywords:** HNF1B, Variants, Prostate cancer, Endometrial cancer

## Abstract

**Background:**

To provide a synopsis of the current understanding of the association between variants of *HNF1B* and cancer susceptibility, we conducted a comprehensive research synopsis and meta-analysis to evaluate associations between *HNF1B* variants and prostate and endometrial cancers.

**Results:**

Eighteen studies totaling 34,937 patients and 55,969 controls were eligible for this meta-analysis. Four variants showed a significant association with the risk of individual cancer. Strong significant associations were found between rs4430796 A and the risk of both prostate cancer (*OR* = 1.247, *p* = 2.21 × 10^− 77^) and endometrial cancer (*OR* = 1.217, *p* = 8.98 × 10^− 16^); the AA, AG genotypes also showed strong significant associations with the risk of prostate cancer (*OR*1 = 1.517, *p* = 4.46 × 10^− 22^; *OR*2 = 1.180, *p* = 0.002). There was a strong significant association between rs7501939 G and the risk of prostate cancer (*OR* = 1.201, *p* = 9.31 × 10^− 31^). Strong significant association was found between rs11649743 G (*OR* = 1.138, *p* = 1.08 × 10^− 12^), rs3760511 C (*OR* = 1.214, *p* = 1.57 × 10^− 19^) and the prostate cancer risk;the GG, AG genotypes of rs11649743 also showed strong significant associations with the risk of prostate cancer (*OR*1 = 1.496, *p* = 3.32 × 10^− 6^; *OR*2 = 1.276, *p* = 7.82 × 10^− 6^). All the cumulative epidemiological evidence of associations was graded as strong.

**Conclusions:**

Our study summarizes the evidence and helps to reveal that common variants of *HNF1B* are associated with risk of prostate and endometrial cancer.

## Background

Human cancers result in Considerable morbidity and mortality. Family history, ethnicity, lifestyle and region are potential risk factors for cancer development [1–4]. However, family-based and adoption studies have provided major evidence for the role of genes in the development of cancers [5–7].

Owing to advances in sequencing technologies and genome-wide association studies (GWAS), a large number of genetic variants correlated with various cancers have been identified [8, 9]. Multiple studies have examined the relationship between the hepatocyte nuclear factor-1 beta (*HNF1B*, formerly known as TCF2) locus (on chromosome 17q12) and cancer risk [10–13]. HNF1B is a member of the homeodomain-containing superfamily of transcription factors and is involved in the tissue-specific regulation of many genes expressed in various organs [14] and during embryonic development [15]. Patients with a heterozygous *HNF1B* deletion exhibit renal disease, elevated liver enzymes, and diabetes [16]. HNF1B is strongly associated with the risks of many cancers, including prostate cancer [10, 17],ovarian cancer [18–20],endometrial cancer [12, 21, 22] and lung cancer [13]. Recently, it has been reported that the rs7501939 single-nucleotide polymorphism (SNP) in *HNF1B* confers a poor overall survival in patients with multiple myeloma [23].

However, fine-mapping studies have revealed a complex genetic architecture of the HNF1B locus, demonstrating that variants of *HNF1B* and the direction of their effects differ between cancer types. SNPs rs4430796 and rs7501939, are both associated with the prostate cancer risk across many ethnic groups [24]. The same SNPs, are also associated with endometrial cancer risk in women of European background [12]. Yet, the SNP rs757210, in high linkage disequilibrium with rs4430796, is the most strongly associated with serous epithelial ovarian cancer [18].

Here, we collected data related to the associations between *HNF1B* variants and cancer phenotypes, and performed a comprehensive meta-analysis, involving a total of 34,937 patients and 55,969 controls, to derive more precise estimates of the associations between *HNF1B* variants and susceptibility to prostate and endometrial cancers.

## Methods

### Search strategy and inclusion criteria

The US National Library of Medicine’s PubMed, Embase, OMIM, ISI Web of Science, and Chinese National Knowledge Infrastructure (CNKI) databases were searched in a systematic manner to retrieve all genetic association studies of *HNF1B* variants and cancers published before July 2017. The search strategy was based on a combination of the terms (Hepatocyte nuclear factor-1 beta or *HNF1B*) and (cancers or tumors). The references of all computer-identified publications were searched for additional studies, and the PubMed option “Related Articles” was also used to search for potentially relevant papers. Searches were performed by two independent reviewers (Yu Tong and Yibin Wang). The language of the publications did not influence article selections.

Studies were included if they met the following criteria. (1) the study reported original data from case-control or cohort studies, (2) the study reported alleles and genotypes for *HNF1B* variants, and (3) the numbers of subjects possessing each allele and genotype in the cancer and control groups were available. No restrictions were set for the source of controls (general population, clinic, or hospital). Studies were excluded when: (i) they lacked sufficient information; (ii) they were published as letters to editors or conference abstracts; (iii) they were studies about cancer mortality.

### Data extraction

Data were extracted independently by two investigators (Yu Tong and Yibin Wang), who used recommended guidelines for reporting on meta-analyses of observational studies. The following data were extracted from the eligible studies: authors, journal title, year of publication, country of origin, selection and characteristics of cases and controls, demographic data, ethnicity of the study population, numbers of eligible and genotyped cases and controls, and genotype distributions in cases, controls, and available subgroups. Furthermore, we examined whether genotype frequencies in control groups conformed to the Hardy-Weinberg equilibrium (HWE) was determined. Any disagreement was adjudicated by a third author (Yi Qu).

### Statistical analysis

The odds ratio was used as the metric of choice for each study. To detect overall genetic associations, allele frequencies were computed for studies reporting allele and genotype data. Pooled odds ratios were computed by the fixed effects model and the random effects model based on heterogeneity estimates. Once an overall gene effect was confirmed, the genetic effects and mode of inheritance were estimated using the genetic model-free approach suggested by *Minelli* et al. We performed Cochran’s Q test and calculated *І*^2^ statistic to evaluate heterogeneity between studies. Harbord’s test was performed to evaluate publication bias. Potential small-study bias was evaluated by Egger’s test [25]. Sensitivity analyses were conducted to examine if the significant association would be lost when the first published report was excluded, or studies deviated from HWE in controls were excluded. All analyses were conducted using Stata, version 14.0 (StataCorp, 2017), with the *metan, metabias, metacum, and metareg* commands.

Venice criteria [26] were applied to evaluate the epidemiological credibility of significant associations identified by meta-analysis. Credibility was defined in three categories: amount of evidence (graded by the sum of test alleles or genotypes among cases and controls: A for > 1000, B for 100–1000, and C for < 100), replication of the association (graded by the heterogeneity statistic: A for *I*^2^ < 25%, B for *I*^2^ between 25 and 50%, and C for *I*^2^ > 50%), and protection from bias (graded as A: there was no observable bias, and bias was unlikely to explain the presence of the association, B: bias could be present, C: bias was evident or was likely to explain the presence of the association, association. C was also assigned to an association with a summary OR less than 1.15, unless the association had been replicated by GWAS or GWAS meta-analysis from collaborative studies. With no evidence of publication bias). Cumulative epidemiological evidence for significant associations was thought to be strong if all three grades were A, moderate if all three grades were A or B, and weak if any grade was C.

To determine whether a significant association could be excluded as a false positive finding, FPRP (false positive report probability) was calculated using the method described by Wacholder et al. [27]. FPRP < 0.05, 0.05 ≤ FPRP ≤0.20, and FPRP > 0.20 were considered strong, moderate, and weak evidence of true association, respectively.

## Results

### Eligible studies

**Fig. 1:**
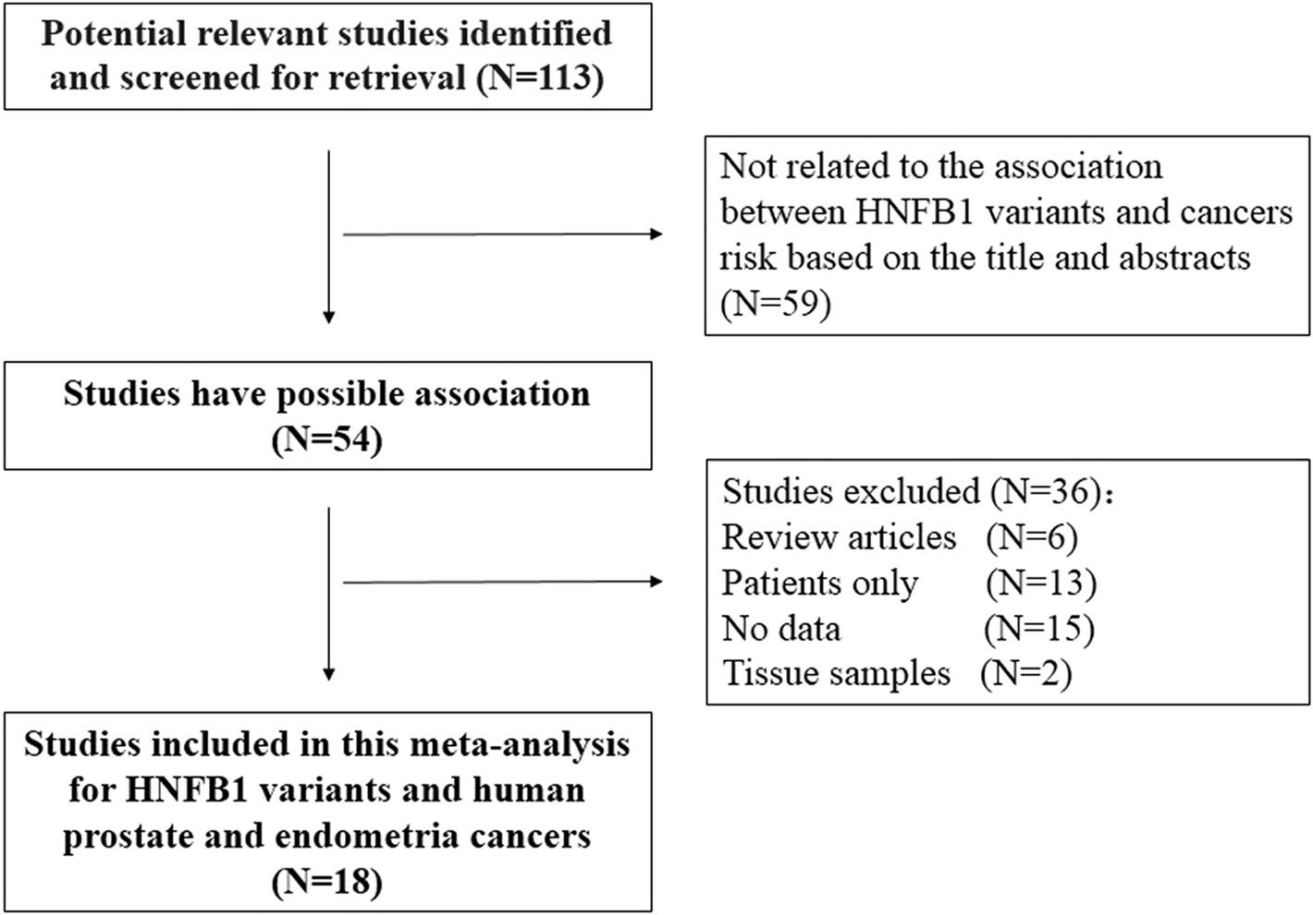
Flow diagram of included and excluded studies

Our initial database search identified 113 potentially relevant studies. Based on a review of titles and abstracts, 55 articles were retained. The full text of these 55 articles was reviewed in detail, and 18 studies containing 36 datasets were eligible for inclusion in the meta-analysis. The specific process for identifying eligible studies and inclusion and exclusion criteria are summarized in Fig. 1a.

Characteristics of the included articles are presented in Allelic associations: 1. Of the 36 datasets, 26 were on prostate cancer [10, 24, 28–41]; and10 were on endometrial cancer [42, 43]. All eligible studies had case-control designs. Cases were recruited from hospital patients and controls were mainly healthy individuals recruited from the hospital or community and were unrelated to cases.

**Table 1:** Characteristics of case-control studies included in a meta-analysis of the association between *HNF1B* variants and human cancers

Ref	Cancer	Region/Center	Ethnicity	rs4430796 cases/ controls	rs7501939 cases/ controls	rs11649743 cases/ controls	rs3760511 cases/ controls
[31]	prostate	China	Asian	195/160			
[32]	prostate	Korean	Asian	240/223	240/223		240/223
[24]	prostate	USA Europe	Caucasian	10,272/9123	10,247/9100	10,272/9123	10,272/9123
[10]	prostate	CAPS	Caucasian	2874/1708		2852/1688	
[10]	prostate	JHH	Caucasian	1521/479		1490/470	
[10]	prostate	ATBC	Caucasian	901/902		927/921	
[10]	prostate	FPCC	Caucasian	620/618		656/656	
[10]	prostate	HPFS	Caucasian	581/591		596/611	
[10]	prostate	PLCO	Caucasian	1121/1048		1166/1093	
[10]	prostate	ACS	Caucasian	1716/1718		1759/1775	
[28]^a^	prostate	Iceland	Caucasian	1501/11289	1501/11289		
[28]	prostate	Netherlands	Caucasian	997/1464	997/1464		
[28]	prostate	Spain	Caucasian	456/1078	456/1078		
[28]	prostate	USA	Caucasian	536/514	536/514		
[29]	prostate	USA	Caucasian	542/473	542/473		
[30]	prostate	USA	Caucasian	1563/576	1563/576		1563/576
[30]	prostate	USA	African	364/353	364/353		364/353
[36]	prostate	Japan	Asian	311/1035			
[40]	prostate	USA	African	454/301	454/301		
[37]	prostate	China	Asian	105/78			
[38]	prostate	Japan	Asian	518/323			
[31]	prostate	USA	Caucasian	754/2713			
[31]	prostate	CGEM	Caucasian	1176/1101			
[39]	prostate	Singapore	Asian	289/141			
[32]	prostate	USA	Caucasian	166/33			
[41]	prostate	USA	Caucasian	759/790			
[42]^a^	endometrial	MEC	Caucasian	106/813	106/813		
[42]	endometrial	WHI	Caucasian	868/3037	868/3037		
[42]	endometrial	MEC	African	68/820	68/820		
[42]	endometrial	WHI	African	35/350	35/350		
[42]	endometrial	MEC	Asian	121/1204	121/1204		
[42]	endometrial	WHI	Asian	8/161	8/161		
[42]	endometrial	MEC	Latino	104/673	104/673		
[42]	endometrial	WHI	Latino	20/207	20/207		
[42]	endometrial	MEC	Hawaiian	27/344	27/344		
[43]	endometrial	Australia and the UK	Caucasian	3048/9528	3048/9528		
Total				34,937/55969	21,305/42508	19,718/16337	12,439/10275

CAPS = CAncer Prostate in Sweden;JHH = The Johns Hopkins Hospital study; ATBC = Beta-Carotene Cancer Prevention Study;FPCC = CeRePP French Prostate Case-Control Study;HPFS = The Health Professionals Follow-up Study;PLCO = Prostate, Lung, Colon and Ovarian (PLCO) Cancer Screening Trial; MEC = Multiethnic Cohort Study; WHI = Women’s Health Initiative; CGEM = Cancer Genetic Markers of Susceptibility Study

^a^Genome-wide association study (GWAS)

### Allelic associations

#### *HNF1B* variants and the risk of prostate cancer

##### rs4430796 G > A and the risk of prostate cancer

**Fig. 2:**
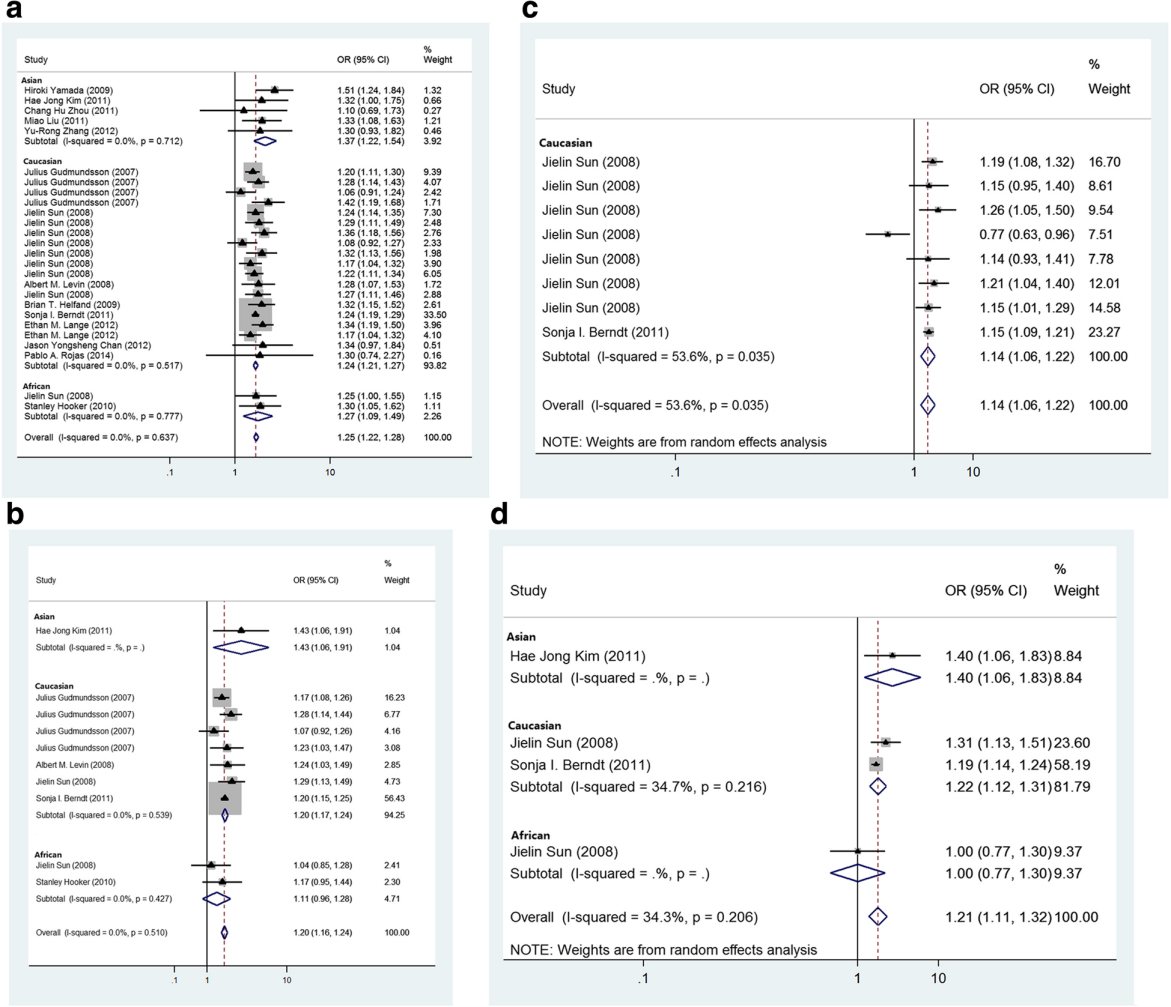
**a** Fixed-effects meta-analysis of allele (A versus G) of the *HNF1B* rs4430796 G > A and prostate cancer. The OR of each study is represented by a square, and the size of the square represents the weight of each study with respect to the overall estimate. 95% CIs are represented by the horizontal lines, and the diamond represents the overall estimate and its 95% CI. **b**. Fixed-effects meta-analysis of allele (G versus A) of the *HNF1B* gene rs7501939 A > G and prostate cancer. **c**. Radom-effects meta-analysis of allele (G versus A) of the *HNF1B* gene rs11649743 A > G and prostate cancer. **d**. Fixed-effects meta-analysis of allele (C versus A) of the *HNF1B* gene rs3760511 A > C and prostate cancer

All 15 publications were included in the evaluation of the association between the *HNF1B* rs4430796 and prostate cancer (Allelic associations: 1). A strong significant association with risk of prostate cancer was observed (*p* = 2.21 × 10^− 77^, fixed effect *OR* = 1.247, 95% *CI*: 1.218, 1.276; *Q* = 21.98, *p* = 0.637, *I*^*2*^ = 0.0%, Fig. 2). Sensitivity analyses in Asians (*p* = 8.32 × 10^− 8^, fixed effect *OR* = 1.369, 95% *CI*: 1.221, 1.536; *Q* = 2.13, *p* = 0.712, *I*^2^ = 0.0%) and Caucasians (*p* = 1.21 × 10^− 69^, fixed effect *OR* = 1.241, 95% *CI*: 1.212, 1.271; *Q* = 17.09, *p* = 0.517, *I*^2^ = 0.0%) demonstrated a pattern similar to that of the full population. However, this effect was weak in the Africans (*p* = 0.002, fixed effect *OR* = 1.275, 95% *CI*: 1.093, 1.487; *Q* = 0.08, *p* = 0.777, *I*^2^ = 0.0%). No publication bias was found in the eligible studies (Harbord’s test *p* = 0.253).

##### rs7501939 A > G and the risk of prostate cancer

Six publications were included in the evaluation of the association between the *HNF1B* rs7501939 and prostate cancer (Table 1). A strong significant association with risk of prostate cancer was observed (*p* = 9.31 × 10^− 31^, fixed effect *OR* = 1.201, 95% *CI*: 1.164, 1.239; *Q* = 8.24, *p* = 0.510, *I*^*2*^ = 0.0%, Fig. 2b). Sensitivity analyses in Caucasians demonstrated a pattern similar to that of the full population (*p* = 1.04 × 10^− 29^, fixed effect *OR* = 1.203, 95% CI: 1.165, 1.242; *Q* = 5.04, *p* = 0.539, *I*^2^ = 0.0%). No publication bias was found in the eligible studies (Harbord’s test *p* = 0.864).

##### rs11649743 A > G and the risk of prostate cancer

Two publications included data regarding the association between the *HNF1B* rs11649743 and prostate cancer (Table 1). There was a significant difference in the between-study heterogeneity among the eligible studies (*Q* = 15.1, *p* = 0.035, *I*^*2*^ = 53.6%). Strong significant association was observed with the prostate cancer risk (*p* = 1.08 × 10^− 12^, random effect *OR* = 1.138, 95% *CI*: 1.062, 1.219, Fig. 2c). No publication bias was found in the eligible studies (Harbord’s test *p* = 0.588).

##### rs3760511 A > C and the risk of prostate cancer

Three publications were included in the evaluation of the association between the *HNF1B* rs3760511 and prostate cancer. There was a strong significant association between rs3760511 and the risk of prostate cancer, and moderate heterogeneity was found among the eligible studies (*p* = 1.57 × 10^− 19^, random effect *OR* = 1.214, 95% *CI*: 1.113, 1.325; *Q* = 4.57, *p* = 0.206, *I*^*2*^ = 34.3%, Fig. 2d). Sensitivity analyses in Caucasians demonstrated a pattern similar to that of the full population (*p* = 6.11 × 10^− 19^, random effect *OR* = 1.216, 95% *CI*: 1.125, 1.314; *Q* = 1.53, *p* = 0.216, *I*^*2*^ = 34.7%). No publication bias was found in the eligible studies (Harbord’s test *p* = 0.778).

### *HNF1B* variants and the risk of endometrial cancer

#### rs4430796 G > A and the risk of endometrial cancer

**Fig. 3:**
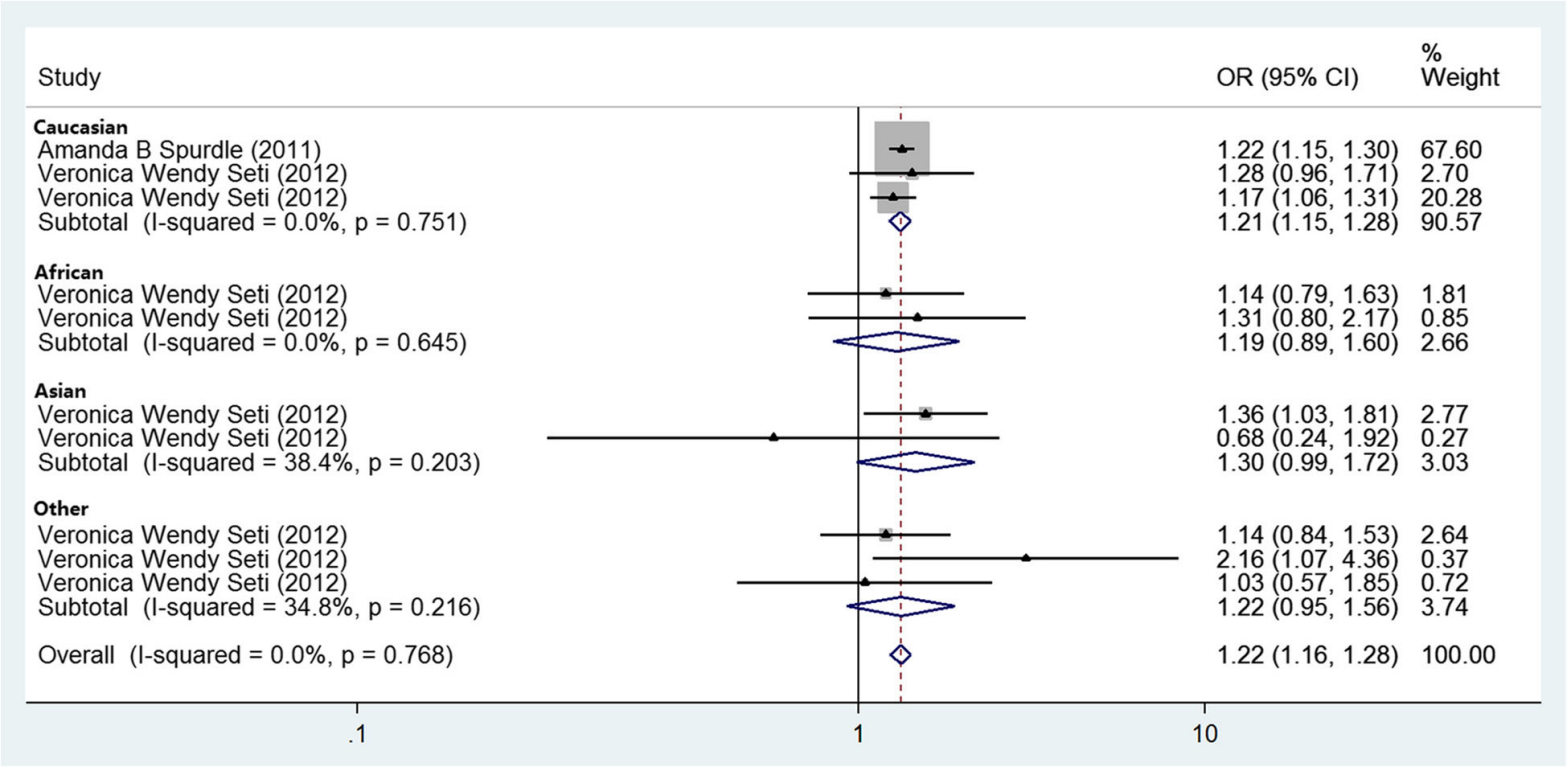
Fixed-effects meta-analysis of allele (A versus G) of the *HNF1B* gene rs4430796 G > A and endometrial cancer. The OR of each study is represented by a square, and the size of the square represents the weight of each study with respect to the overall estimate. 95% CIs are represented by the horizontal lines, and the diamond represents the overall estimate and its 95% CI

Two publications were included in the evaluation of the association between the *HNF1B* rs4430796 A > G and endometrial cancer (Table 1). There was a strong significant association between rs4430796 and the endometrial cancer risk (*p* = 8.98 × 10^− 16^, fixed effect *OR* = 1.217, 95% *CI*: 1.160, 1.276; *Q* = 5.72, *p* = 0.768, *I*^*2*^ = 0.0%, Fig. 3). Similar patterns were found in the Caucasians (*p* = 3.73 × 10^− 14^, fixed effect *OR* = 1.215, 95% *CI*: 1.155, 1.277; *Q* = 0.57, *p* = 0.751, *I*^*2*^ = 0.0%). Lack of significant association was found in Africans (*p* = 0.235, fixed effect *OR* = 1.193, 95% *CI*: 0.891, 1.597; *Q* = 0.21, *p* = 0.645, *I*^*2*^ = 0.0%), the Asians (*p* = 0.058,fixed effect *OR* = 1.304, 95% *CI*: 0.992, 1.716; *Q* = 1.62, *p* = 0.203, *I*^*2*^ = 38.4%), and Latino and Hawaiian (*p* = 0.122, fixed effect *OR* = 1.217, 95% *CI*: 0.949, 1.562; *Q* = 3.07, *p* = 0.216, *I*^*2*^ = 34.8%). No publication bias was found in the eligible studies (Harbord’s test *p* = 0.950).

#### rs7501939 G > A and the risk of endometrial cancer

Two publications were included in the analysis of the association between the *HNF1B* rs7501939 and endometrial cancer (Table 1). Although the risk of endometrial cancer was increased in individuals carrying the G allele, compared to those with the A allele, lack of significant association was found with endometrial cancer risk (*p* = 0.258, random effect *OR* = 1.204, 95% *CI*: 0.873, 1.660). The same pattern was observed in Caucasians (*p* = 0.751, random effect *OR* = 1.104, 95% *CI*: 0.599, 2.036; *Q* = 190.13, *p* = 0.000, *I*^*2*^ = 98.9%), Africans (*p* = 0.122, random effect *OR* = 1.254, 95% *CI*: 0.942, 1.670; *Q* = 0.93, *p* = 0.336, *I*^*2*^ = 0.0%), Asians (*p* = 0.918, random effect *OR* = 1.040, 95% *CI*: 0.492, 2.196; *Q* = 2.23, *p* = 0.136, *I*^*2*^ = 55.1%)and Latino and Hawaiian (*p* = 0.262, random effect *OR* = 1.389, 95% *CI*: 0.783, 2.464; *Q* = 6.28, *p* = 0.043, *I*^*2*^ = 68.2%) (Data not shown).

### Genotype comparison

#### rs4430796 G > A and the risk of prostate cancer

Of the 15 publications, only seven reported genotype information. The genotype distribution of the *HNF1B* rs4430796 among case and control groups is presented in Table 2. The genotype effects for AA versus GG (*OR*1) and AG versus GG (*OR*2) were calculated for each study. A multivariate meta-analysis was conducted to estimate the pooled risk. There was a significantly increased risk of prostate cancer among individuals with the homozygous AA genotype (*p* = 4.46 × 10^− 22^, fixed effect *OR*1 = 1.517, 95% *CI*: 1.394, 1.651; *Q* = 12.27, *p* = 0.424, *I*^*2*^ = 2.2%) and heterozygous AG genotype (*p* = 0.002, random effect *OR*2 = 1.180, 95% *CI*: 1.064, 1.309; *Q* = 17.50, *p* = 0.132, *I*^*2*^ = 31.4%).The pooled estimates were similar to those obtained after removal of the study with HW disequilibrium [10], i.e., fixed effect *OR*1 = 1.524 (*p* = 7.97 × 10^− 18^,95% *CI*: 1.384, 1.677; *Q* = 12.23, *p* = 0.347, *I*^*2*^ = 10.1%) and random effect *OR*2 = 1.198 (*p* = 0.003,95% *CI*: 1.064, 1.348;*Q* = 16.43, *p* = 0.126, *I*^*2*^ = 33.1%).

**Table 2:** The association between the *HNF1B* rs4430796 and prostate cancer (genotype distribution of case-control studies included in a meta-analysis)

Ref	Cases			Controls			HWE	AA vs GG	AG vs GG
	GG	AG	AA	GG	AG	AA		OR1 (95% CI)	OR2 (95% CI)
[31]	16	60	119	77	73	10	0.889	1.494(1.249–1.786)	1.087(0.920–1.285)
[10]	446	1355	1073	316	883	509	^a^0.025	1.697(1.255-2.296)	1.285(0.984–1.679)
[10]	254	779	488	106	253	120	0.155	1.955(1.441–2.653)	1.433(1.060–1.937)
[10]	87	395	419	136	431	335	0.445	1.190(0.869–1.631)	1.077(0.820–1.415)
[10]	149	308	163	161	309	148	0.495	1.756(1.264–2.441)	1.304(0.974–1.746)
[10]	113	289	179	153	300	138	0.349	1.332(1.052–1.688)	0.998(0.808–1.233)
[10]	254	522	345	257	529	262	0.378	1.445(1.196–1.747)	1.206(1.018–1.428)
[10]	357	843	516	434	850	434	0.332	1.806(1.351–2.413)	1.543(1.187–2.006)
[37]	12	34	59	6	34	38	0.335	0.776(0.269–2.244)	0.500(0.168–1.486)
[38]	52	214	252	45	149	129	0.425	1.691(1.076–2.656)	1.243(0.792–1.950)
[39]	21	99	169	11	63	67	0.235	0.966(0.417–2.238)	0.514(0.217–1.215)
[32]	11	75	80	4	15	14	0.498	1.321(0.604–2.889)	0.823(0.372–1.823)
[41]	240	390	129	198	388	204	0.310	2.078(0.579–7.455)	1.818(0.510–6.484)
Pooled								1.517(1.394–1.651)	1.180(1.064–1.309)

HWE = *p*-value for Hardy–Weinberg equilibrium;

^a^Hardy–Weinberg disequilibrium was observed in the control group

#### rs11649743 A > G and the risk of prostate cancer

Only one publication reported genotype information for rs11649743. However, this publication included relevant data for different populations and regions. The genotype distribution for the *HNF1B* rs11649743 among case and control groups is presented in Table 3. The genotype effects for GG versus AA (*OR*1) and GA versus AA (*OR*2) were calculated for each study. Multivariate meta-analysis was conducted to estimate the pooled risk. There was a significantly increased risk of prostate cancer among individuals with the homozygous GG genotype (*p* = 3.32 × 10^− 6^, fixed effect *OR*1 = 1.496, 95% *CI*: 1.262, 1.772) and heterozygous AG genotype (*p* = 7.82 × 10^− 6^, fixed effect *OR*2 = 1.276, 95% *CI*: 1.072, 1.519). No between-study heterogeneity was found for the homozygous GG genotype (*Q* = 2.19, *p* = 0.902, *I*^*2*^ = 0.0%) or for the heterozygous GA genotype (*Q* = 2.30, *p* = 0.891, *I*^*2*^ = 0.0%).

**Table 3:** Association between the *HNF1B* rs11649743 and prostate cancer (genotype distribution of case-control studies included in the meta-analysis)

Ref	Cases			Controls			HWE	GG vs AA	GA vs AA
	AA	GA	GG	AA	GA	GG		OR1 (95% CI)	OR2 (95% CI)
[10]	115	895	1842	90	587	1009	0.292	1.460 (1.099–1.941)	1.220 (0.910–1.635)
[10]	40	395	1055	14	139	317	0.396	1.165 (0.626–2.168)	0.995 (0.525–1.884)
[10]	18	219	690	27	250	644	0.324	1.607 (0.877–2.946)	1.314 (0.704–2.451)
[10]	20	191	445	32	211	413	0.227	1.724 (0.971–3.062)	1.448 (0.801–2.618)
[10]	19	159	418	27	174	410	0.063	1.449 (0.793–2.646)	1.299 (0.695–2.426)
[10]	28	361	777	47	359	687	0.200	1.898 (1.176–3.065)	1.688 (1.034–2.756)
[10]	48	495	1216	62	546	1167	0.425	1.346 (0.916–1.979)	1.171 (0.788–1.740)
Pooled								1.496 (1.262–1.772)	1.276 (1.072–1.519)

### Cumulative evidence of association

#### Epidemiological credibility of significant associations

Venice criteria were applied to evaluate these significant associations. Details of protection from bias for genetic variants significantly associated with prostate and endometrial cancer risk in meta-analyses are shown in Table 4. Grades of A were given to all these meta-analyses for amount of evidence, replication of association, and protection from bias. Therefore, strong evidence of true association with cancer risk is assigned to rs4430796, rs7501939, rs11649743, and rs3760511 for prostate cancer and rs4430796 for endometrial cancer.

**Table 4:** Details of protection from bias for genetic variants significantly associated with prostate and endometrial cancers risk in meta-analyses

Variants	Cancer site	Cancer risk		Venice criteria grade	Protection from bias	Reason for bias	Reason for bias exemption	Initial study influence		Deviation from HWE	OR < 1.15	*p* value for publication bias	*p* value for small study bias
		OR (95% CI)	*p* value					OR (95% CI)	*p* value				
rs4430796	prostate	1.247 (1.218–1.276)	2.21 × 10^−77^	AAA	A	NA	Identified by GWAS	1.244 (1.215–1.273)	2.07 × 10^−74^	No	No	0.253	0.248
rs7501939	prostate	1.201 (1.164–1.239)	9.31 × 10^−31^	AAA	A	NA	Identified by GWAS	1.200 (1.162–1.238)	1.31 × 10^−29^	No	No	0.864	0.868
rs11649743	prostate	1.138 (1.062–1.219)	1.08 × 10^−12^	AAA	A	Low OR	Identified by GWAS	1.136(1.053–1.226)	0.001	No	Yes	0.588	0.580
rs3760511	prostate	1.214 (1.113–1.325)	1.57 × 10^−19^	AAA	A	NA	Identified by GWAS	1.228 (1.139–1.224)	1.04 × 10^−7^	No	No	0.778	0.770
rs4430796	endometrium	1.217 (1.160–1.276)	8.98 × 10^−16^	AAA	A	NA	Identified by GWAS	1.202 (1.105–1.308)	1.87 × 10^−5^	No	No	0.950	0.943

HWE = *P* value for Hardy-Weinberg equilibrium;

#### Probability of true association with cancer risk

To evaluate the probability of true association with cancer risk for the nominally significant variants, FPR*P* value was calculated. All associations with cancer risk had a FPRP value < 0.001. Thus, all the cumulative epidemiological evidence of associations was graded as strong.

## Discussion

To our knowledge, this is the first general overview of the association between *HNF1B* variants and susceptibility to prostate and endometrial cancers. Our primary analysis revealed that, rs4430796 A, showed strong significant associations with risk of both prostate cancer (*OR* = 1.247, *p* = 2.21 × 10^− 77^,) and endometrial cancer (*OR* = 1.217, *p* = 8.98 × 10^− 16^); the AA, AG genotypes also showed strong significant associations with risk of prostate cancer (*OR*1 = 1.517, *p* = 4.46 × 10^− 22^; *OR*2 = 1.180, *p* = 0.002). Sensitivity analyses in Caucasians demonstrated patterns similar to that of the full population. However, lack of significant association was found in Africans, which is likely due to the considerably smaller sample size. There was a strong significant association between rs7501939 A and the risk of prostate cancer (*OR* = 1.201, *p* = 9.31 × 10^− 31^); however, lack of significant association with endometrial cancer risk was observed (*OR* = 1.104, *p* = 0.751. For rs11649743 G, strong significant association was found with the prostate cancer risk (*OR* = 1.138, *p* = 1.08 × 10^− 12^), and the GG, AG genotypes also showed strong significant associations with the risk of prostate cancer (*OR*1 = 1.496, *p* = 3.32 × 10^− 6^; *OR*2 = 1.276, *p* = 7.82 × 10^− 6^). Strong significant association was also found between rs3760511 C and the risk of prostate cancer (*OR* = 1.214, *p* = 1.57 × 10^− 19^). Using the Venice criteria and false-positive report probability tests, we graded all the cumulative evidence of significant associations with prostate and endometrial cancers risk as strong.

Our findings were based on several gene-association studies, including several thousand participants, and were robust in terms of study design and sensitivity analyses. We found no evidence of publication bias or small study bias based on funnel plots. Between-study heterogeneity was found in allelic association studies (G versus A) of rs7501939, and in allelic (G versus A) of rs11649743 for prostate cancer. When HWE was examined, one study showed deviation. Our results were robust to the removal of this study.

*HNF1B* encodes three isoforms: isoforms (A, B and C); isoform A and B act as transcriptional activators and isoform C acts as a transcriptional repressor [44]. HNF1B is involved in the regulation of cell proliferation, and genetic variation in *HNF1B* might modulate the risk of cancer [45]. However, the precise pathomechanism by which the genetic variation affects susceptibility to cancers is still unclear. In a recent GWAS, rs4430796 and rs7501939 in *HNF1B* were associated with the risks of both endometrial cancer in women of European background [43] and prostate cancer [28] . Several studies examined the associations between HNF1B and prostate cancer and endometrial cancer across various populations [12, 46, 47]. According to these studies, the two variants are associated with the risks of prostate cancer and endometrial cancer. Moreover, the rs4430796 G allele is significantly associated with an increased risk of lung cancer [13] . In 2013, Pharoah et al. identified that the *HNF1B* rs757210 is specific to serous epithelial ovarian cancer by pooling data from GWAS and follow-up genotyping; the analysis included 43 studies from the Ovarian Cancer Association Consortium [18]. At the same time, Shen et al. found evidence for a differential effect of HNF1B on the serious and clear cell subtypes of ovarian cancer. They found that HNF1B loss-of-function role and gain-of-function are related to serous and clear cell ovarian cancers, respectively [20]. Another research discovered *HNF1B* rs7501939 was a susceptibility locus for testicular germ cell tumor [48]. Taken together, these studies suggest that specific *HNF1B* variants predispose individuals to clear cell ovarian, endometrial, lung and prostate cancers, et al.

There are several limitations of the study. First, it is likely that some publications were overlooked although we conducted an exhaustive literature search, some relevant published studies with null results were not identified. Second, due to insufficient data, we were unable to evaluate publication bias for associations between several variants in 8q24 region and prostate and endometrial cancer. Third, a unified analysis standard across studies could not be defined for lack of raw data from the original publications. Therefore, future studies with larger sample size are warranted to confirm these associations.

## Conclusions

Given the relevance of *HNF1B* variants to cancer biology, we attempted to estimate the strength of the genetic associations between these variants and prostate and endometrial cancers. This Human Genome Epidemiology (HuGE) systematic review presents strong evidence for an association between *HNF1B* variants and prostate and endometrial cancers, both overall and in Caucasians, Asians, Africans, and Indians, suggesting a multiplicative genetic model for variants of *HNF1B* among different ethnic populations. Our study results also suggest that *HNF1B* plays an important role in prostate and endometrial cancers, and these variations may serve as efficient and economical biomarkers for the diagnosis of prostate and endometrial cancers.

## Abbreviations

CNKI: Chinese National Knowledge Infrastructure
GWAS: genome-wide association studies
HNF1B: hepatocyte nuclear factor-1 beta
SNP: single-nucleotide polymorphism
